# Protective Effect of Epigallocatechin-3-Gallate (EGCG) in Diseases with Uncontrolled Immune Activation: Could Such a Scenario Be Helpful to Counteract COVID-19?

**DOI:** 10.3390/ijms21145171

**Published:** 2020-07-21

**Authors:** Marta Menegazzi, Rachele Campagnari, Mariarita Bertoldi, Rosalia Crupi, Rosanna Di Paola, Salvatore Cuzzocrea

**Affiliations:** 1 Department of Neuroscience, Biomedicine and Movement Sciences, Biochemistry Section, School of Medicine, University of Verona, Strada Le Grazie 8, I-37134 Verona, Italy; rachele.campagnari@univr.it (R.C.); mita.bertoldi@univr.it (M.B.); 2 Department of Veterinary Science, University of Messina, Polo Universitario dell’Annunziata, I-98168 Messina, Italy; rcrupi@unime.it; 3 Department of Chemical, Biological, Pharmaceutical and Environmental Sciences, University of Messina, Viale Ferdinando Stagno D’Alcontres 31, I-98166 Messina, Italy; dipaolar@unime.it (R.D.P.); salvator@unime.it (S.C.); 4 Department of Pharmacological and Physiological Science, Saint Louis University School of Medicine, St. Louis, MO 63104, USA

**Keywords:** green tea extract, EGCG, COVID-19, cytokines, JAK/STAT, NF-κB, rheumatoid arthritis, multiple sclerosis, inflammatory bowel diseases, antiviral activity

## Abstract

Some coronavirus disease 2019 (COVID-19) patients develop acute pneumonia which can result in a cytokine storm syndrome in response to Severe Acute Respiratory Syndrome coronavirus 2 (SARS-CoV-2) infection. The most effective anti-inflammatory drugs employed so far in severe COVID-19 belong to the cytokine-directed biological agents, widely used in the management of many autoimmune diseases. In this paper we analyze the efficacy of epigallocatechin 3-gallate (EGCG), the most abundant ingredient in green tea leaves and a well-known antioxidant, in counteracting autoimmune diseases, which are dominated by a massive cytokines production. Indeed, many studies registered that EGCG inhibits signal transducer and activator of transcription (STAT)1/3 and nuclear factor kappa-light-chain-enhancer of activated B cells (NF-κB) transcription factors, whose activities are crucial in a multiplicity of downstream pro-inflammatory signaling pathways. Importantly, the safety of EGCG/green tea extract supplementation is well documented in many clinical trials, as discussed in this review. Since EGCG can restore the natural immunological homeostasis in many different autoimmune diseases, we propose here a supplementation therapy with EGCG in COVID-19 patients. Besides some antiviral and anti-sepsis actions, the major EGCG benefits lie in its anti-fibrotic effect and in the ability to simultaneously downregulate expression and signaling of many inflammatory mediators. In conclusion, EGCG can be considered a potential safe natural supplement to counteract hyper-inflammation growing in COVID-19.

## 1. Introduction

The new emerging coronavirus disease 2019 (COVID-19), caused by Severe Acute Respiratory Syndrome coronavirus 2 (SARS-CoV-2) infection, is characterized by a wide spectrum of symptoms and clinical course from asymptomatic form to mild or serious illness. Unfortunately, a subset of patients develops an acute respiratory distress syndrome (ARDS) consisting of an intense pneumonia associated with a systemic immune response that is able to compromise organ functions and frequently leads to fatal event [1]. The pathogenetic mechanisms involved in COVID-19 aggravation have not been deeply understood in these few months since its first appearance. Anyway, ARDS in COVID-19 looks like those of two other previous coronavirus epidemics, Severe Acute Respiratory Syndrome (SARS) and Middle East Respiratory Syndrome (MERS) in which the abnormal host immune response has been recognized as the main determinant leading to adverse outcome. Indeed, in COVID-19, very critical patients display a cytokine storm syndrome with an increased secretion of interleukin (IL)-6, IL-1β, IL-17, IL-8, tumor necrosis factor (TNF)-α, interferon (IFN)-γ and other pro-inflammatory mediators [1,2]. This over-production of pro-inflammatory cytokines induces more damage to the host cells than the one induced by SARS-CoV-2 as pathogen invader. Besides the high cytokine release, a relevant worsening of systemic functionality has been registered, with a reduction of total circulating lymphocytes, infiltration of monocytes/macrophages and neutrophils in the lungs, vasculitis with hypercoagulability, increase of C-reactive protein, transaminases, ferritin, and creatinine serum levels. All these features point out that multiple organ damages have occurred [3]. Hence, it is evident that either prevention or resolution of hyper-inflammation are crucial determinants to avoid mortality in COVID-19.

As currently no specific therapies are available and the management of the disease is only supportive, the entire research community is looking at other better-known syndromes in which such a scenario looks like that of ARDS in COVID-19. Indeed, observational data have shown overlapping clinical features with some autoimmune diseases characterized by a massive release of cytokines.

Secondary hemophagocytic lymphohistiocytosis (sHLH), also called macrophage activation syndrome (MAS), is known as severe disorder characterized by intense hyper-immune response ultimately leading to a cytokine storm. sHLH/MAS has been described by the presence of several signs of immune-dysfunction, such as (i) natural killer cells inability to clear antigen after infection or autoimmune stimulation; (ii) persistence of antigen presentation; (iii) repression of regulatory T (Treg) cell functionality; (iv) activation and proliferation of tissue macrophages (histiocytes); and (v) secretion of pro-inflammatory cytokines by activated histiocytes, which induces systemic hyper-inflammatory syndrome [4,5]. Clinical features and laboratory data of sHLH include sustained fever, splenomegaly, hyper-ferritinemia, pancytopenia, fibrinolytic consumptive coagulopathy and liver dysfunction [6], which are very close to those described in severe COVID-19. sHLH can arise as a result of infectious or autoimmune diseases. Epstein-Barr virus and cytomegalovirus, most frequently, or influence and dengue infections, to a lesser extent, can trigger sHLH [7]. Among the autoimmune diseases, sHLH derives principally from complication of systemic juvenile idiopathic arthritis or its adult equivalent, adult onset Still disease, even though it can also occur in other systemic inflammatory disorders i.e., systemic lupus erythematosus, Kawasaki disease, and periodic fever syndromes [5,6]. Besides sHLH, similarities between severe COVID-19 and other more common autoimmune diseases, in which naive T helper (Th) cells preferentially differentiate in Th1 and Th17 subsets rather than Th2 and Treg cells have also been documented. Actually, abnormal activation of Th1 and Th17 cells leads to increased secretion of IFN-γ, IL-2, TNF-α, IL-1β, IL-17, IL-21, and IL-6, which triggers inflammation. In addition, a reduced functionality of Treg helps to keep the inflammatory response turned on [8]. Rheumatoid arthritis, Sjogren’s syndrome, multiple sclerosis, and inflammatory bowel diseases present that peculiarity.

## 2. Conventional and Biological Therapies in Autoimmune Diseases and COVID-19

For a long time, corticosteroid drugs have been largely used in autoimmune diseases management for their role as remission inducers and their broad spectrum anti-inflammatory action [9]. The main effects of glucocorticoids are to inhibit several pro-inflammatory genes encoding cytokines, chemokines, cell adhesion molecules, and enzymes to address the inflammatory process and restore homeostasis. Although their efficacy in suppressing inflammation is well recognized, corticosteroid drugs display many adverse events, including high risk of developing comorbidities, especially infections [10]. Thus, the results of clinical studies on the role of corticosteroids in infectious diseases showing inflammatory hallmarks is still controversial. Corticosteroids have been widely used also during SARS and MERS coronavirus disease outbreaks. A recent meta-analysis study, examining SARS, MERS trials and the first results on COVID-19 clinical experimentation, reported that treatment with corticosteroids raised the mortality rate and reduced virus clearance [11], although a final response is still missing.

Drugs counteracting the biological effects of cytokines are now largely used in rheumatology and in other autoimmune diseases and they have demonstrated efficacy. Anti-IL-1, anti-TNF-α, and anti-IL-6 monoclonal antibodies can deplete each specific target slowing down the cytokine storm. Tocilizumab, an IL-6 receptor blocker, has been used to manage autoimmune diseases, indeed, it has been approved for the treatment of a variety of clinical conditions that display cytokine release syndrome [9], and it is having promising results also in severe COVID-19 [12]. Baricitinib is an oral drug used in rheumatoid arthritis treatment. It functions as a blocker of Janus kinases (JAK), the upstream activating enzymes of the signal transducers and activators of transcription (STATs) involved in type I/II IFNs and IL-6 signaling [13]. The use of JAK inhibitors is attractive because they are well tolerated, and give the opportunity to target numerous inflammatory cytokine signaling pathways simultaneously [14,15].

In conclusion, biological drugs, blocking specific cytokine effects, are repurposed to counteract the inflammatory host response triggered by SARS-CoV-2 [16]. Actually, many clinical trials are undergoing to prove their efficacy.

## 3. Epigallocatechin-3-Gallate (EGCG) in Autoimmune Diseases

EGCG, the most abundant (approximately 50%) and active catechin present in the green tea extract (GTE), displays a wide range of beneficial effects including anti-inflammatory, anticarcinogenic, antimicrobial, and immunomodulating effects [17,18].

### 3.1. Rheumatoid Arthritis

Rheumatoid arthritis (RA) is an autoimmune disease with persistent inflammation of synovial joints which can lead to articular bone and cartilage disruption [19].

Haqqi et al., firstly reported a protective effect of green tea polyphenols (0.2% GTE solution in drinking water) in a collagen-induced arthritis (CIA) mouse model of RA in which a reduced incidence of arthritis and a downregulation of inflammatory mediators, i.e., IFN-γ, TNF-α, cyclooxygenase 2, were attested [20]. Amelioration of adjuvant-induced mouse arthritis was reported by Ahmed et al. who showed how EGCG (100 mg/kg, by intraperitoneal injection (i.p)., administrated daily from day 7 to day 16) can inhibit IL-6 synthesis and suppress its signaling by producing the soluble form of gp130 IL-6-co-receptor [21]. GTE added in the drinking water (8 g/L, 2/week) reduced RA symptoms also in a rat model of adjuvant-induced arthritis showing a decrease of IL17 and a concomitant increase of IL-10 serum level, suggesting that Th17 and Treg cell subsets can be regulated in the opposite way by these polyphenols [22]. It must be reminded here that high Th17/Treg ratio is a hallmark of RA [23]. Hence, molecules that are able to restore the balance between Th17 and Treg could be crucial tools for RA management.

The mechanism of Th17 reduction was investigated in a CIA mouse model, in which the EGCG treatment (40 mg/kg, 3/week, i.p.) was able to decrease the number of phospho-STAT3 positive T cells [24]. Similar data were reported by Brun et al. who attested the suppression of both STAT3 activity and Th17 cell differentiation (20 mg/kg of EGCG, 3/week, i.p.) [25]. In addition, Lee et al. further confirmed the capability of EGCG (20–50 mg/kg, 3/week, i.p.) to downregulate Th17 clonal expansion, suppress STAT3 activity, but they also registered an increase of extracellular signal-regulated kinase (ERK) phosphorylation, nuclear factor erythroid 2-related factor 2 (Nrf-2) activity, and heme oxygenase-1 (HO-1) expression [26]. Notably, Th17 clonal expansion needs STAT3-dependent-IL6 signaling [27], thus, experimental data obtained by these groups are in line with the reduction of IL-6 expression and signaling previously reported by Ahmed et al. [21]. 

Finally, to evaluate the risk factor on RA onset, it must be highlighted the result of a prospective cohort study including 30,000 old women of Iowa (USA) without a history of the disease. Women consuming >3 cups/day of tea displayed an inverse association risk of RA onset [28]. Even though this is a promising result, further clinical experimentations are required to prove the preventive and therapeutic effects of EGCG in human RA.

### 3.2. Sjogren’s Syndrome

Sjogren’s syndrome (SS) is a relatively common autoimmune disease characterized by lacrimal and salivary glands inflammation. In SS, autoantigen expression and apoptotic cell death are important etiological factors leading to loss of secretory function [29,30]. EGCG can protect autoimmune-induced distress of salivary glands in non-obese diabetic mouse model of SS syndrome. EGCG administration (0.1–0.2% EGCG solution in drinking water) reduced lymphocytic infiltration in the glands, as well as inhibited apoptosis and cell proliferation [29]. Hsu et al. demonstrated that GTE (0.2% GTE solution in drinking water) can decrease autoantibody level in animal serum, and showed that EGCG can prevent the TNF-α-induced cytotoxicity in ex vivo salivary gland cells [30]. Saito et al. used an autoimmune sialadenitis model of MRL-Fas-lpr mice in which oxidative damage triggers apoptosis in salivary gland cells. Damaged glands displayed a reduced expression of water channel aquaporin 5 (AQP5), resulting in a low exocrine flow of saliva following pilocarpine stimulation. EGCG (592 µg/mouse in drinking water, for 57 days) was able to restore AQP5 expression level, finally improving gland functionality. The EGCG-protective action (2 and 10 mg/kg in drinking water, for ten days) was mediated by the inactivation of both nuclear factor kappa-light-chain-enhancer of activated B cells (NF-κB) and c-Jun N-terminal kinase, and by the preservation of protein kinase A activity [31]. All data suggest that EGCG supplementation can be efficacious also in a little but well-defined area of the body in which inflammatory damage had occurred.

### 3.3. Multiple Sclerosis

Multiple sclerosis (MS) is a T-cell dependent autoimmune disease involving nervous system and displaying inflammation, demyelination, axonal injury, and gliosis [18]. A well-characterized rodent model for human MS is the experimental autoimmune encephalomyelitis (EAE), in which the disease is induced by animal immunization with myelin derived proteins. Similarly to MS, EAE histopathology showed signs of perivascular inflammatory lesions and demyelination in the central nervous system (CNS) [8].

Neuroprotective attribute of EGCG is well-known for its ability to increase antioxidant enzymes activities, such as superoxide dismutase and catalase, in the brain [32]. Aktas et al. firstly demonstrated in a EAE model that EGCG (100 µL/mouse in saline, 3/die, by oral gavage) suppressed brain inflammation and reduced symptoms and neuronal damages [33]. These protective effects were mediated by inhibition of both NF-κB activity and expression of its target gene TNF-α [33]. In this regard, we highlighted that NF-κB is a transcription factor crucial for MS pathogenesis given that it regulates genes encoding inflammatory mediators and plays key roles in resident cells of the CNS during disease development [34]. Wang et al. confirmed in EAE mice that EGCG, dose dependently (0.15–0.6%, orally, for 30 days), ameliorated clinical symptoms, delayed diseases onset, reduced inflammatory infiltration and demyelination damage [35]. Mechanistically, EGCG altered the balance between T cell subsets, reducing Th1 and Th17 pro-inflammatory T cells and promoting Treg cell population with the consequent decrease in IFN-γ and IL17 production. Since low level of IL6 expression is crucial to redirect transforming growth factor-β (TGF-β)-induced naïve T cells to Treg subtype instead of Th17 one, IL6 secretion has been measured. Data showed that EGCG inhibited IL6 production, as well as, both ICAM1 expression and leukocytes infiltration in the CNS, accordingly with the noted role of adhesion molecules to regulate immune-cell migration across the blood–brain barrier [35]. Importantly, as the EAE improvement took place even if the EGCG was administered 7-12 days after immunization, the authors concluded that the EGCG effect is largely therapeutic rather than preventive and, therefore, EGCG could be suitable for MS therapy.

Moreover, several studies promoted the association between EGCG (300 µg, 2/die, by oral gavage, for 50 days) and other natural products, such as glatiramer acetate in EAE model [36,37], or coconut oil in MS patients [38]. In all these studies synergistic effects of the combination therapy were registered. EGCG and glatiramer acetate can improve the expression of both brain-derived neurotrophic factor (BDNF) and beneficial cytokines in CNS [36], and promote an increase in HO-1 expression level [37], whereas, in MS patients, EGCG (800 mg/die, orally, for 4 months) plus coconut oil improved anxiety and disability by decreasing IL6 serum level [38]. The clinical trials using EGCG in MS patients are shown in Table 1.

In conclusion, the therapeutic effects of EGCG in MS are promising. The beneficial impact was supported by its multiple molecular target activity and its good CNS bioavailability.

### 3.4. Inflammatory Bowel Diseases

Crohn’s disease and ulcerative colitis are chronic inflammatory bowel diseases (IBD) mediated by immune dysregulation [39]. The IL-1β, IL-6, IL-8, TNF-α, and IFN-γ unbalance drives to chronic inflammatory status that interferes with the homeostasis of the gastrointestinal tract finally leading to diarrhea, bloating, gas, bleeding, and abdominal pain [40]. In addition, IBD are characterized by oxidative and nitrative stress, leukocyte infiltration, and up-regulation of intercellular adhesion molecule 1 (ICAM-1) expression in the colon [41].

Several studies have been performed to prove whether EGCG administration could be helpful in the IBD. Human T-cell line Kit 225, similarly to primary Th17 cell subtype, can secrete IL-17 and other pro-inflammatory cytokines, including TNF-α. In either unstimulated or IL-23 stimulated T-cell Kit 225, EGCG treatment inhibited IL-17 and TNF-α expression [42]. The protective effect of green tea polyphenols was confirmed by an acetic acid-induced colitis model, in which EGCG (50 mg/kg/die, by oral gavage) improved rat mucosal inflammation by drastically decreasing TNF-α, IFN-γ, and NF-κB expression levels and reducing nitric oxide (NO) production and malondialdehyde activity [43]. In a different rat model of experimental colitis, induced by intracolonic instillation of dinitrobenzene sulphonic acid, we showed that treatment with GTE (50 mg/kg/die, i.p.) significantly attenuated diarrhea and loss of body weight. These effects were associated with a remarkable improvement of the disrupted colonic architecture, with significant reduction of myeloperoxidase and TNF-α production. GTE also reduced both the appearance of nitro-tyrosine tissue immunoreactivity and the expression of ICAM-1 [41]. IL-2 knockout C57BL/6 mice develop a colon inflammation comparable to the human ulcerative colitis, in which the cytokine profile is consistent with a Th1-type host immune response leading to an increase of IFN-γ and TNF-α expression levels [44]. Lamina propria lymphocytes were isolated from colon of IL-2 KO mice treated or not with green tea polyphenols, and then cultured ex vivo. GTE (5 g/L in drinking water) markedly reduced lymphocytes IFN-γ production. Authors commented that green tea polyphenols, that are able to modulate Th1-mediated response in the gut, are likely to be of significant clinical relevance in the treatment of Crohn’s disease which displays Th1 feature [44]. In another type of experimental colon injury, dextran sodium sulfate administration can induce erosion and increases permeability in colonic epithelium, which results in clinical signs of ulcerative colitis. In this model, EGCG treatment (20 and 50 mg/kg/die, by oral gavage) prevented colon shortening, and improved both intestinal permeability and histopathological changes. In particular, the EGCG-elicited attenuation of colonic lesions was accompanied by IL-6, MCP-1, TNF-α suppression and inhibition of macrophage infiltration [45]. In the same animal model, using both wild type and IL-10 deficient rats, Oz et al. reported that EGCG (0.12–0.5%/die incorporated into daily diet) significantly attenuated the colitis severity in a comparable way to sulfasalazine [46]. The authors concluded that, as sulfasalazine, commonly used in IBD therapy, has serious adverse effects, these promising results suggest that EGCG or GTE could become an alternative safe therapy for human IBD.

## 4. EGCG Molecular Mechanisms that Could Counteract COVID-19 Hyper-Inflammation

In many experimental models of acute inflammation, we proved the anti-inflammatory effects of polyphenols present in the GTE [41,47,48,49,50]. In particular, in a mouse model of carrageenan-induced pleurisy, we reported that GTE (25 mg/kg, i.p.) attenuated lung injury by lowering ICAM-1 and TNF-α expression, STAT-1 activation, nitrotyrosin and polyADP-ribosyl protein modifications, and polymorphonuclear leukocytes (PMNs) accumulation in the lungs [47]. We also used an animal model of multiple organ dysfunction syndrome (MODS) characterized by lung injury followed by hepatic, intestinal, renal, and cardiac dysfunctions. MODS results in the simultaneous trigger of complement, coagulation, and fibrinolytic cascades which induce a generalized inflammatory response. The latter leads to the activation of phagocytes and endothelial cells and release of many cytokines and other pro-inflammatory mediators [51]. MODS displays similar features to that of ARDS in COVID-19. In this model, triggered by zymosan administration in mice, we provided evidence that GTE reduces (i) development of zymosan-induced peritonitis, (ii) PMNs infiltration in the lung and intestine, (iii) renal dysfunction and (iv) liver, lung, pancreatic and intestinal injury [48].

The molecular mechanisms underlined the protective effects of GTE have been investigated in in vitro experiments on human epithelial cell lines, including A549 alveolar and DLD1 colon cells. We demonstrated the capability of EGCG, at 5–20 µM range, and GTE at 4–40 µg/mL range, to strongly inhibit IFN-γ-triggered STAT1 activation. EGCG/GTE exerted their effect toward the inhibition of JAK2-elicited STAT1 phosphorylation, leading to the blockade of the expression of target genes i.e., inducible nitric oxide synthase, chemokine (C-X-C motif) ligand (CXCL)9, CXCL10, ICAM1, and the class II major histocompatibility complex HLA-DR [52,53,54]. The anti-inflammatory action of EGCG was not mediated by its antioxidant activity because *N*-acetyl-cysteine and ascorbate were ineffective [54]. 

IFNs are crucial cytokines in inflammation and in pathogen infection diseases. They belong to different classes with either overlapped or specific functions. Extracellular secreted type I IFNs (IFN-α/IFN-β) play the first battle against viral infection. Actually, type I IFNs, secreted from infected cells, bind to their transmembrane receptors activating STAT1 and STAT2 and trigger IFN-stimulated gene (ISG) expression which rapidly generates a cellular antiviral state [55]. Whereas, type II IFN-γ is an important mediator of immunity and inflammation, since it plays a crucial role in macrophage activation, autoimmunity and Th1 response. IFN-γ signal takes advantage of STAT1 pathway to achieve transcriptional activation of IFN-γ-inducible genes, some of them encoding pro-inflammatory cytokines and chemokines [56]. Nevertheless, viruses can blind the immune system inducing an inadequate host response. Indeed, SARS-CoV failed to induce competent IFN response in SARS patients [57]. Even though the IFN response to SARS-CoV-2 infection is not well characterized, the first important experimental data are now emerging. Angiotensin-converting enzyme 2 (ACE2) is a cellular receptor which allows SARS-CoV-2 internalization in the host cell. Ziegler et al. identified ACE2 as an interferon-stimulated gene. Indeed, in the primary basal cells of nasal mucosa a significant induction of ACE2 was found after IFN-α2 and, to a lesser extent, IFN-β or IFN-γ stimulation [58]. Accordingly, ACE2, in human but not in mouse, must be considered a canonical ISG for the presence of STAT1, STAT3, IRF8, and IRF1 binding sites on its promoter [58]. Another fundamental study analyzed the bronchoalveolar lavage fluid (BALF) from eight COVID-19 patients in comparison with either healthy controls or non-COVID-19 pneumonia patients [59]. A distinct pattern of gene expression has been found in SARS-CoV-2 infected patients with respect to healthy subjects as well as to those of SARS-CoV or other pathogen-induced pneumonia patients. Data derived from the SARS-CoV and MERS-CoV outbreaks have revealed that these coronaviruses suppress type-I IFN response by interfering with Janus kinases/signal transducers and activators of transcription (JAK/STAT) signaling [16]. On the contrary, SARS-CoV-2 triggers a robust IFN response by increasing the expression of several ISGs [59]. Nevertheless, the protective potential of some ISGs failed to inhibit virus replication, perhaps for the above described ACE2 super-induction, instead an aggravation of lung inflammation occurred. In this regard, by a genomic analysis at the BALF cells of COVID-19 patients, Zhou et al. showed a high enrichment of ISG members belonging to the inflammatory regulation cluster which included neutrophil-recruiting mediators CXCL8, CXCL1, CXCL2, CXCL10, chemokine (C-C motif) ligand (CCL)2, CCL7 and other attractants of monocytes and immune cells CXCL6, CXCL11, CCL2, CCL3, CCL4, CCL7, CCL8, as well as STAT1 itself which, by positive feedback, leads to the persistence of the inflammatory status [59]. In COVID-19 patients, all data suggest a preponderance of pro-inflammatory purpose in the response to IFNs instead to develop antiviral reaction [59]. This could be deleterious because, in that way, IFNs can support the cytokine storm. In agreement with these data, Major et al. described a mechanism by which type I and III IFNs signaling aggravated lung pathology in coronavirus-infected mice [60]. Moreover, Broggi et al. reported that type III IFN-λ, produced by lung dendritic cells following viral recognition, induced barrier damage by inhibiting lung epithelial repair [61]. All these results provide a strong rational for using molecules that are able to block JAK/STAT activity, such as JAK inhibitors, in COVID-19 therapy. Indeed, many clinical trials are ongoing to test JAK inhibitors efficacy (i.e., tofacitinib, NCT 04412252, NCT04415151, NCT04332042; baricitinib, NCT04390464, NCT04393051, NCT04373044, NCT04321993, NCT04345289, NCT04320277, NCT04340232; ruxolitinib, NCT04362137, NCT04414098, NCT04331665, NCT04377620, NCT04338958, NCT04334044, NCT04348071). To further examine the clinical trials listed above, we recall Table 1 presented in the review of Luo et al. [15]. The advantage of JAK/STAT pathway inhibition should reside in the blockade of several cytokines and chemokines synthesis and secretion [62]. Thus, JAK/STAT inhibitors, both in autoimmune diseases [62] and in COVID-19 [15], are attractive therapeutic tools to abrogate signaling pathway versus multiple targets at the same time. EGCG/GTE, at low micromolar concentration in in vitro and in preclinical studies, act as STAT1 inhibitors like the synthetic JAK inhibitors. 

Several studies supported a broad anti-inflammatory action of EGCG and GTE which cannot be limited to scavenging toxic oxidants and to inhibit STAT-1 activity. As already reported, STAT3 activation was also reduced by EGCG and GTE administration [24,25,26]. Similarly to STAT1 [53], the molecular mechanism involved in the STAT3 inhibition was elucidated by surface plasmon resonance (SPR) assay and by in silico docking model [63]. SPR assay indicated that EGCG significantly interrupted STAT3 peptide binding at micromolar concentrations, and docking experiments indicated that EGCG competitively binds to the STAT3 SH2 domain, inhibiting STAT3 phosphorylation and signaling [63]. Importantly, in the immune system, most of the cytokine receptors, including the receptors of IL-6, IL-10, IL-21, and IL-23, can activate STAT3 indicating that it is an important nuclear factor for the regulation of immune responses and autoimmunity [64]. It is also known that in COVID-19 hyper-inflammatory condition, high level of IL-6 seem to be the main prognostic factor for worse outcomes. Indeed, the use of drugs that are able to inhibit IL-6 signaling, such as tocilizumab, can block the progression of disease [12] and, in a similar way, EGCG could be promising too because it is a powerful blocker of STAT3 pathway.

EGCG/GTE can also prevent the activation of NF-κB [31,33,43,65,66,67,68,69], the main architect of inflammatory signals for its key role in numerous immunologic processes. NF-κB controls the expression of many pro-inflammatory cytokines, including IL-1β, TNF-α, IL8, IL-6, all of which are induced in the cytokines storm syndrome [70,71] and in COVID-19 [72,73].

The anti-inflammatory action of EGCG/GTE also derives by their ability to activate Nrf2 nuclear translocation and HO-1 activity leading to protective effects, in particular, on neuronal cells [74], in arthritis [75], and in atherosclerosis [76].

Thus, EGCG/GTE should be repurposed in COVID-19 with the aim to revert the hyper-inflammatory status. 

## 5. EGCG Is Protective against Lung Fibrosis

We also discuss the importance to target lung fibrosis. A substantial proportion of patients who develop ARDS die for the progressive pulmonary fibrosis. Important fibrosis mediators include matrix metalloproteinases, associated with vascular endothelial growth factor (VEGF) and cytokines release, that induce epithelial and endothelial injury [77]. The pulmonary fibrosis in COVID-19 patients occurs because SARS-CoV-2 infection induces massive increase of neutrophils infiltration into the lungs, with the production and the activation of TGF-β. Uncontrolled increase in active TGF-β, with the help of proinflammatory cytokines such as TNFα, IL-6, and IL-1β, results in rapid and massive edema and fibrosis that remodels and ultimately blocks the airways, finally leading to the lung functional failure [78]. Indeed, the risk of poor outcome is higher in fibrotic lung disease following SARS-CoV-2 infection than in not-fibrotic one. In addition, the global burden of fibrotic lung disease increases considerably with age, male sex, and comorbidities such as hypertension and diabetes [79]. Therefore, there is an urgent need for therapies that mitigate lung fibrosis in severe COVID-19 [79].

It must be reminded the protective effects of EGCG/GTE in different models of lung fibrosis. Idiopathic pulmonary fibrosis (IPF) is a lethal chronic progressive pulmonary disease and the TNF-alpha transgenic mouse is an animal model for human IPF. These mice overexpress TNF-alpha only in the lungs and have been used to verify the protective effect of GTE. The TNF-α overexpression was continuous, IL-1α and IL-1β were overexpressed only in the early stage, while IL-6 production increased along with the progression of interstitial pneumonia. These data suggest that IL-6 secretion contributed, together with TNF-α, to the development of the disease [80]. After treatment with GTE, mRNA and protein levels of TNF-α and IL-6 were reduced by 70% and 80%, respectively, suggesting that green tea has significant preventive effects on the TNF-α-related diseases [80]. Moreover, beneficial efficacy of EGCG (20 mg/kg, i.p., for 28 days) was reported against a bleomycin-induced rat model of lung fibrosis, in which lysosomal hydrolases and ultrastructural changes in the lungs were improved [81]. In the same model, Sriram et al. showed that EGCG (20 mg/kg, i.p., for 28 days) supplementation reduced NF-κB activity, TNF-α, IL-1β expression and induced Nrf2 signaling [82]. In addition, EGCG inhibited fibroblast activation and collagen accumulation by downregulating TGF-β1 signaling [83]. In conclusion, EGCG/GTE can be considered as potent anti-fibrotic agents.

## 6. EGCG/GTE Studies for Many Clinical Applications

Several clinical trials have been performed to test the safety and the advantages of EGCG supplementation in either prevention or therapeutic use (Table 1).

EGCG has emerged as a chemopreventive product with anticancer activity for its ability to target several oncogenic signaling pathways and it has been recently tested in various phases of clinical trials [84]. EGCG in the Polyphenon E preparation was well tolerated by patients with chronic lymphocytic leukemia in a phase II trial. Durable declines in the total lymphocytic count and lymphadenopathy were observed in the majority of patients [85]. EGCG clinical trials have been conducted targeting postmenopausal women with high risk of developing breast cancer. EGCG can afford benefit in terms of regulating LDL-cholesterol, glucose and insulin, as reported by a double-blind, randomized, placebo-controlled interventional study in healthy postmenopausal women [86]. A systematic review of the literature for the ability of EGCG to lower low-density lipoprotein cholesterol (LDL-C) was performed. Data showed that consumption of green tea EGCG resulted in a significant reduction of LDL-C and the effect size was slightly dependent on the baseline lipid level of the subjects [87]. A double-blind, randomized, placebo-controlled trial further confirmed the benefit of EGCG on blood lipids in healthy postmenopausal women [88]. Finally, a meta-analysis was done to evaluate the association between green tea intake and risk of cardiovascular diseases or ischemic related diseases. The study provides evidence that consumption of green tea is associated with favorable outcomes [89].

All studies did not report significant adverse effects and registered the ability of EGCG/GTE to restore natural homeostasis in many different pathologies. Given their low toxicity risk and the large consumption, EGCG and GTE have potential use as safe natural supplement either for prevention or for treatment of several diseases with inflammation hallmarks.

**Figure 1:**
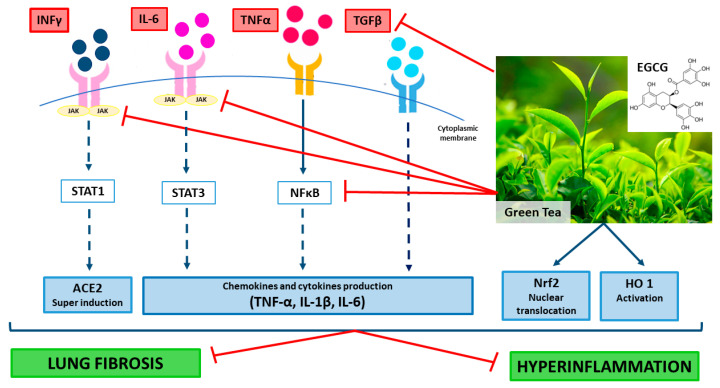
Mechanisms of the potential beneficial effects of green tea extract and epigallocatechin-3-gallate (EGCG) in coronavirus disease 2019 (COVID-19) patients.

In conclusion, in COVID-19 the EGCG/GTE supplementation should be advantageous because of their multitarget action as regulators of both transcription factor (i.e., STAT1, STAT3, NF-κB, Nrf2) activities and expression of their target genes (Figure 1). EGCG/GTE could restore the tissue homeostasis counteracting the pro-inflammatory action of IFNs and cytokines and the onset of lung fibrosis (Figure 1).

## 7. Protective Effect of GTE/EGCG in Experimental Sepsis

Sepsis is a systemic inflammation syndrome due to a dysregulated host response to bacterial or viral infection, characterized by excessive accumulation of inflammatory mediators and impairment to restore homeostasis. Tissue damage is partially elicited by pathogen- and damage-associated molecular patterns. Among them the high mobility group box 1 (HMGB1), that is released by activated monocytes/macrophages and functions as a late mediator of endotoxemia and sepsis, plays a key role [93]. Indeed, mice administration of recombinant HMGB results in clinical signs of sepsis and, conversely, antibodies anti-HMGB1 or its inhibitors protect mice against lipopolysaccharide (LPS)-induced acute tissue injury and lethal sepsis [93,94]. Instead, selective deletion of IL-1β, IL-1R type 1, IL-18, and inhibitor κB kinase β, as well as, loss of NLR family pyrin domain containing 3 (NLRP3), a canonical inflammasome component, fails to promote survival in experimental sepsis or induce severe immunodeficiency [95]. In this context, it should be highlighted the importance of selectively targeting damage-mediated inflammation, specially HMGB1, whilst maintaining the physiological protective immune responses [95].

It has been known for a long time that GTE confers protection against lethality in a murine model of endotoxin-induced sepsis [96] and that EGCG administration, (10 mg/kg, i.p.), improves polymicrobial rat sepsis by inhibiting both NF-κB activation and inducible NO synthase (iNOS) expression [97]. Data of Wang et al. recently confirmed a protective effect of 10 mg/kg administration of EGCG in acute LPS-induced lung mice injury, showing an associated reduction of TNF-α, IL-1β, IL-6, Toll-like receptor-4 levels, and NF-κB activation [98]. Importantly, EGCG promoted significant long-lasting protection against experimental sepsis as 4 mg/kg (i.p.) delayed administration of this catechin, i.e., 24 or 48 h after cecal ligation and puncture (CLP), significantly rescued mice from sepsis, thus supporting a therapeutic potential of EGCG in clinical management of human septicemia [99]. Li et al. demonstrated again that EGCG attenuates IL-6 and HMGB1 serum levels in mice after CLP, as well as, 10 µM EGCG reduces the secretion of IL-6, TNF-α and nitric oxide in primary murine peritoneal macrophages that were stimulated with HMGB1 [99]. Finally, a pro-autophagic activity of EGCG (at 2–20 µM range) in an endotoxin stimulated macrophages model was also registered, resulting in autophagic HMGB1 degradation and protection against endotoxemia [100]. Another potential molecular mechanism could explain the EGCG ability to decrease HMGB1 serum level. Notably, JAK/STAT1 may represent a critical signaling mechanism controlling HMGB1 translocation from nucleus to cytoplasm before its secretion [95]. In both lethal endotoxemia and experimental sepsis inhibition of the JAK/STAT1 pathway by genetic deletion of STAT1 [101], or inhibition of IFN-β expression by knockout of IRF3 [102], significantly decrease HMGB1 release and enhance animals survival [95,103]. As already reported above, we demonstrated that EGCG inhibits STAT1 activation both in vitro and in pre-clinical studies [47,50,52,53,54]. Hence, we suggest that the EGCG inhibitory effect on HMGB1 secretion could be mediated by the catechin ability to block STAT1 activity with a consequently hindrance of HMGB1 cytosolic translocation. 

Sepsis is a common feature of COVID-19, that is often associated with sepsis-induced coagulopathy leading to disseminated intravascular coagulation and resulting in high related mortality [104]. Importantly, the overproduction in serum of two alarmins S100A8/A9 and HMGB1 in patients with COVID-19 was associated with distinct signatures for cytokine storm. Serum levels of these parameters are of great clinical significance and can be utilized to identify COVID-19 patients with poor outcomes [105]. In agreement with these results, some researchers recently proposed that HMGB1 should be considered a crucial therapeutic target in COVID-19 therapy [106,107].

Together, these findings indicate that targeting HMGB1 may be beneficial and that GTE/EGCG can be also useful in both prevention and decrease of sepsis by counteracting its deleterious effects with several mechanisms, as already suggested by Wyganowska-Swiatkowska et al. [108].

## 8. Antiviral Activity of EGCG and Other Green Tea Polyphenols

Likewise to their antibacterial properties, green tea polyphenols are known to possess antiviral activities against a wide range of DNA and RNA viruses. Among natural catechins, EGCG was found to be the most potent virus inhibitor and the 3-galloyl and 5′-OH groups appear crucial for this activity [109,110]. In particular, EGCG at the micromolar concentration inhibits the infectivity of herpes simplex virus (HSV), hepatitis C virus (HCV), influenza A virus, human immunodeficiency virus (HIV), Zika virus, dengue virus and many others [111]. The broad antiviral properties of EGCG are due to its high affinity but nonspecific binding to viral surface proteins, since EGCG competes with heparan sulfate or sialic acid for virions first attachment [111,112]. The more stable form of EGCG (EGCG-palmitoyl ester) is 8–24 times more effective than native EGCG to block H1N1 influenza virus, Ebola virus and HSV-2, with a long-lasting protective action (up to 48 h) [113]. By the way, EGCG-palmitate is currently used as ingredient in different products for its generalized increase in antimicrobial efficacy [113]. It is worth noting that green tea catechins are effective in prophylaxis for influenza infections in humans, as emerging by clinical trials [114,115], in addition to in vitro results [116]. Thus, EGCG and other green tea polyphenols could be excellent candidates as non-toxic agents that can prevent a broad range of human and animal viral infections [113].

On the other hand, green tea catechins can block the virus’ life cycle in infected cells. As it concerns HIV-AIDS, EGCG can prevent HIV replication in human peripheral blood cells in vitro by blocking the HIV-1 reverse transcriptase [117,118]. Additionally, EGCG can downregulate the expression of CD4 receptor and reduce HIV viral DNA-integrase binding [112,119]. Moreover, EGCG inhibits serine protease activity of HCV with IC_50_ of 8.5 µM [120], as well as, HCV cell-to-cell spread [121].

It must also be reminded that FDA has approved Veregen, a catechin-derivative drug, for topical treatment of papilloma virus genital lesions (Food and Drug Administration 2006 http://www.accessdata.fda.gov/drugsatfda_docs/nda/2006/021902s000TOC.cfm).

Coronaviruses (CoVs) are positive-sense, single-stranded RNA viruses that infect a wide range of animal hosts. Like SARS and MERS CoVs, SARS-CoV-2 genome contains ORF1a and ORF1ab open reading frames which codify for two peptides required for viral replication cycle [122]. The proteolytic processing of these peptides is one of the crucial steps in the life cycle of CoVs, which encode a papain-like protease and 3-chymotrypsin-like protease (3-CL^pro^) [123,124].

Chen et al. reported that theaflavin-3,3′-digallate, but not EGCG, is an inhibitor of the main SARS-CoV protease 3CL^Pro^, with an IC_50_ of 7 µM [123]. Instead, Nguyen et al. showed that quercetin, EGCG and gallocatechin gallate (GCG), with IC_50_ in the 50–80 µM range, displayed inhibitory activity in vitro on the SARS-CoV-3CL^pro^ expressed in *Pichia pastoris* [125]. Recently, Khan et al. carried out a molecular docking of seven SARS-CoV-2 proteins with 18 hypothetical inhibitors which had been previously recognized to be anti-SARS-CoV agents [126]. The authors reported that EGCG displays a strong molecular interaction with all SARS-CoV-2 proteins tested, including 3CL^Pro^. In this in silico study, EGCG was found to be even more active than the approved drugs for anti-SARS-CoV-2 therapy Remdesivir and Chloroquine [126]. Likewise, Bhardwaj et al., by molecular dynamics simulation, reported that other tea extract components, such as oolonghomobisflavan-A, theasinensin-D and theaflavin-3-o-gallate, have higher docking score than the repurposed antiviral drugs, Atanazavir, Lopinavir and Darunavir, used in COVID-19 therapy [127]. Moreover, Lung et al. found that theaflavin, that is derived by catechins oxidation and is principally present in black tea, has a lower binding energy when it docks in the catalytic pocket of SARS-CoV-2 RNA-dependent RNA polymerase [128]. Finally, Ghosh et al. showed that EGCG, epicatechin gallate and GCG display strong interaction with His41 and/or Cys145 of the catalytic pocket of SARS-CoV-2-3CL^Pro^, so these catechins can be potential 3CL^Pro^ inhibitors [129]. In summary, the active molecules present in green tea extract form a greater number of hydrogen bonds than the complexes with repurposed antiviral drugs, suggesting strong interaction and stability with important viral enzymes [127]. Despite high expectations, it should be recalled here that these promising results must be validated in both cell and animal models, before considering green tea polyphenols efficacious anti SARS-CoV-2 agents. Since green tea catechins and, specially, EGCG present no toxicity and good human intestine absorption, these studies give the rational to set up in vitro and in vivo experiments for the development of new antiviral drugs against COVID-19.

## 9. Conclusion Remarks

Considering all the properties and the safety profile of EGCG and/or GTE in human, we can speculate that catechins supplementation will be at least partially effective in controlling the inflammation damages that occur in SARS-CoV-2 infection. It should be reminded that, until now, there is no direct evidence in favor of the hypothesis that EGCG treatment could improve COVID-19 outcome. The limitation of our hypothesis lies on the fact that the results reported in this review come from other infections or diseases with uncontrolled immune activation. Anyway, its effectiveness against COVID-19 should be evaluated. Thus, we propose to set up a clinical trial in COVID-19 using EGCG, in addition to antiviral or other anti-inflammatory drugs, as a multitasked anti-inflammatory agent aimed at limiting exacerbated cytokines release. Taking into account that a timely administration of EGCG to COVID-19 patients is most likely crucial, we suggest administering EGCG, orally, at the dosage of 600–900 mg/die, once the symptoms aggravate and/or the blood C-reactive protein, or other markers of inflammation, increase. We expect that EGCG administration can prevent the further aggravation leading to inflammatory markers decline. Finally, it must be reminded that EGCG treatment should also improve coagulopathy-associated to sepsis and lung fibrosis. 

## Figures and Tables

**Table 1:** Clinical Trials on Epigallocatechin-Gallate.

Title	Status	Trial Number	Treatment	Results	Reference
Normalization of dyrk1A and APP Function as an Approach to Improve Cognitive Performance and Decelerate AD Progression in Down Syndrome Subjects: Epigallocatechin Gallate as Therapeutic Tool	Completed	NCT01699711	A daily oral dose containing 9 mg/kg (range 6.9–12.7) of epigallocatechin 3-gallate (EGCG) is given for 12 months	Improving visual recognition memory, inhibitory control, and adaptive behavior	[90]
Effects of EGCG (Epigallocatechin Gallate) in Huntington’s Disease (ETON-Study) (ETON)	Completed	NCT01357681	Maximal daily dose of 1200 mg during a period of 12 months	Not posted	
Effect of Epigallocatechin-Gallate on Inner Retinal Function in Ocular Hypertension and Early Glaucoma	Completed	NCT00476138	(200 mg/day) for 3 months	Modest beneficial effect of EGCG supplementation on inner retinal function	[91]
Sunphenon EGCG (Epigallocatechin-Gallate) in the Early Stage of Alzheimer’s Disease (SUN-AK)	Completed	NCT00951834	Months 1–3: 200 mg EGCG/die Months 4–6: 400 mg EGCG/die Months 7–9: 600 mg EGCG/die Months 10–18: 800 mg EGCG/die	Not posted	
Impact of Ketone Bodies and Epigallocatechin Gallate in Multiple Sclerosis	Completed	NCT03740295	600 mg of epigallocatechin gallate (EGCG) and 60 ml of coconut oil (3600 mg of TGCM) per day, divided into two doses (one in the morning and one at noon)	Reduction of IL-6 level accompanied by an improved state of anxiety and functional capability	[38]
Epigallocatechin Gallate Lowers Circulating Catecholamine Concentrations and Alters Lipid Metabolism	Completed	NCT03199430		Not posted	
Effect of Green Tea (Epigallocatechin Gallate) on Albuminuria in Patients with Diabetic Nephropathy	Completed	NCT01923597	200 mg/capsule Administered orally 4 capsules per day for 3 months	Reduction of podocyte apoptosis and attenuation of residual albuminuria	[92]
Chemopreventive Effects of Epigallocatechin Gallate (EGCG) in Colorectal Cancer (CRC) Patients	In progress	NCT02891538	450 mg PO twice a day	Not posted	
Sunphenon Epigallocatechin-Gallate (EGCg) in Duchenne Muscular Dystrophy (SUNIMUD)	Completed	NCT01183767	10 mg/kg body weight	Not posted	
Epigallocatechin Gallate (EGCG) to Improve Cognitive Performance in Foetal Alcohol Syndrome (FAS) Children (Neuro-SAF)	Completed	NCT02558933	9 mg/kg/day for 1 year	Not posted	
Sunphenon in progressive forms of Multiple sclerosis (SUPREMES)	Completed	NCT00799890	200/800 mg	Not posted	
Effect of green tea intervention on lipoprotein cholesterol, glucose and hormones levels in healthy postmenopausal women	Completed		400 mg or 800 mg/day administered orally for 2 months	Low density lipoprotein (LDL)-cholesterol, glucose and insulin decreased	[86]
Phase 2 trial of daily, oral polyphenon E in patients with asymptomatic, Rai stage 0-II chronic lymphocytic leukemia (CLL)	Completed		Polyphenon E 2000 mg twice daily for up to 6 months	Absolute lymphocyte count (ALC) was reduced	[85]
